# Extremely Low-Frequency Magnetic Exposure Appears to Have No Effect on Pathogenesis of Alzheimer’s Disease in Aluminum-Overloaded Rat

**DOI:** 10.1371/journal.pone.0071087

**Published:** 2013-08-12

**Authors:** Cheng Zhang, Yue Li, Chao Wang, Ruili Lv, Tao Song

**Affiliations:** Beijing Key Laboratory of Bioelectromagnetism, Institute of Electrical Engineering, Chinese Academy of Sciences, Beijing, China; Alexander Flemming Biomedical Sciences Research Center, Greece

## Abstract

**Objective:**

Extremely low-frequency magnetic field (ELF-MF) has been reported to be of potential pathogenetic relevance to Alzheimer's disease (AD) for years. However, evidence confirming this function remains inconclusive. Chronic Al treatment has been identified as a contributing factor to cognitive function impairment in AD. This study aims to examine whether or not ELF-MF and Al have synergistic effects toward AD pathogenesis by investigating the effects of ELF-MF with or without chronic Al treatment on SD rats.

**Methods:**

Sprague-Dawley (SD) rats were subjected one of the following treatments: sham (control group), oral Al (Al group), ELF-MF (100 µT at 50 Hz) with oral Al (MF+Al group), or ELF-MF (100 µT at 50 Hz) without oral Al (MF group).

**Results:**

After 12 wk of treatment, oral Al treatment groups (Al and MF+Al groups) showed learning and memory impairment as well as morphological hallmarks, including neuronal cell loss and high density of amyloid-β (Aβ) in the hippocampus and cerebral cortex. ELF-MF without Al treatment showed no significant effect on AD pathogenesis. ELF-MF+Al treatment induced no more damage than Al treatment did.

**Conclusions:**

Our results showed no evidence of any association between ELF-MF exposure (100 µT at 50 Hz) and AD, and ELF-MF exposure does not influence the pathogenesis of AD induced by Al overload.

## Introduction

Alzheimer's disease (AD) is the most common form of dementia among older people. No single factor has yet been identified as its direct cause. A combination of factors, including age, genetic inheritance, environmental and dietary factors, may be possibly responsible for AD development [1]–[5].

Studies investigating the risk of Alzheimer’s disease and environmental factors have focused on extremely low-frequency magnetic field (ELF-MF) exposure (associated with power lines, transformer substations, *etc*.) in recent years [6], [7]. An initial study regarding the relationship between AD and ELF-MF was reported by Sobel *et al.* in 1995 [7], this study suggested that increased risks of AD could be related to ELF-MF exposure based on the combined results of three other independent studies. The finding was immediately followed by a large number of studies, some of which supported the relationship between AD and ELF-MF exposure [8], [9] and some of which failed to confirm this supposed relationship [10], [11]. Three other investigations provided mixed evidence: Based on a case-control study, Seidler *et al.* [6] suggested that exposure to ELF-MF was not significantly associated with AD dementia; Qiu *et al.* [12] evaluated lifetime occupational exposures in a community cohort of individuals 75 years older and showed marked effects in males but not in females; and Andel *et al.* showed that moderate (≥0.12 to <0.20 µT) to high (≥0.20 µT) levels of MF exposure were associated with AD [13]. As the contradictory and inconclusive epidemiologic results, World Health Organization published Environmental Health Criteria (EHC NO. 238) of extremely low frequency fields in 2007, which suggested there was only inadequate evidence to show that 50/60 Hz fields could cause Alzheimer's disease, and more investigations were further needed [14].

A recent investigation regarding the effects of high frequency electromagnetic field (EMF) on mice AD was reported by Arendash [15]. The results showed that long-term high-frequency EMF exposure, which is directly associated with cell phone use (918 MHz; 0.25 W/kg), has both cognitive-protective and cognitive-enhancing effects on normal mice as well as transgenic mice predisposed to developing Alzheimer’s-like cognitive impairment. However, to date, few controlled long-term studies have been published regarding ELF-MF effects on AD in animal models.

Al toxicity was first recognized in patients to whom Al was administered through renal dialysates [16]. Numerous studies have begun to suggest a possible link between Al intake and AD pathogenesis. Animals loaded with Al developed symptoms and brain lesions similar to those found in AD [17]–[19]. Al may also be involved in other neurodegenerative disorders, such as amyotrophic lateral sclerosis and Parkinsonism-dementia [19]. ELF-MF and Al exist widely in our environment. Mankind was exposed to Al through food, drinking water or other sources. The human who lives next to the power lines and transformer substations may exposure to both ELF-MF and Al simultaneously. Moreover, evidence that chronic exposure to ELF-MF alters blood-brain barrier (BBB) permeability in diabetic rats has been published [20], [21]. Al ions have been reported to cross the BBB through various mechanisms, including transferrin and other carriers [22], thereby increasing BBB permeability and exposing the brain to excess Al accumulation [23]. Potential increases in BBB permeability induced by ELF-MF exposure may result in excess accumulation of Al and induce brain lesions. In this study, the effects of ELF-MF (100 µT at 50 Hz) on the pathogenesis of AD in Al-overdosed rat were investigated.

## Materials and Methods

### Animals and Groups

Adult male Sprague-Dawley (SD) rats aged 10 wk (weight 250 g to 300 g) were purchased from Beijing HFK Bio-Technology Co., Ltd. (Beijing, China). The rats were randomly assigned to four groups: control group rats (n = 10) were placed in a geomagnetic environment and received ultrapure water; Al group rats (n = 10) rats were placed in a geomagnetic environment and fed with AlCl_3_ solution; MF group rats (n = 10) were exposed to a magnetic field (100 µT at 50 Hz) and given free access to ultrapure water; MF+Al 263+2.group rats (n = 10) were exposed to magnetic field (100 µT at 50 Hz) and fed with AlCl_3_ solution. The exposure period was 12 wk, and the experiments were repeated three times.

### Ethics Statement

Animal experimental procedures were performed according to the National Guidelines for Experimental Animal Welfare (Ministry of Science and Technology of People’s Republic of China, 2006) and approved by the Animal Welfare Committee of Beijing Key Laboratory of Bioelectromagnetism. All attempts were made to minimize the number of animals used and avoid undue sufferings.

### Aluminum Treatment

All rats were allowed unlimited access to standard solid food (Beijing HFK Bio-Technology Co., Ltd.) and ultrapure water or AlCl_3_ solution for 12 consecutive weeks. The AlCl_3_ used was of analytical-reagent grade and obtained from Aldrich Chemical Co. (Milwaukee, Wisconsin, USA). The ultrapure water (18.2 MΩ·cm) used during the experiments was obtained by ultra-filtrating the distilled water through a Milli-Q purification system (Milli-Q Academic A10, Massachusetts, USA). The AlCl_3_ solution was prepared by dissolving 2 g of AlCl_3_ in 1 L of ultrapure water. Water intake was recorded at about 10 AM every day, and body weight was recorded every week during the experiment.

### Exposure System and Field Characteristics

The rats were housed in plastic cages in an air-conditioned room with a constant temperature of 23°C±1 and kept in a 12 h: 12 h light-dark cycle (lights on from 8 AM to 8 PM). Rats in the MF and MF+Al groups were kept in an exposure region with a magnetic flux density of 100 µT (rms) generated by an MF exposure apparatus fabricated in our laboratory. The apparatus consisted of a pair of Helmholtz coils (diameter: 1400 mm) and an alternating current power source (50 Hz). The magnetic flux density was measured using an EM field analyzer (EFA-300, Wandel and Goltermann, NC, USA), and the magnetic field direction was vertical. Rats in the control and Al groups were placed in a geomagnetic environment in the sham region, in which the ambient magnetic field (50 Hz) was less than 400 nT. Non-magnetic supports were used to place plastic drinking bottles in the cages.

**Figure 1:**
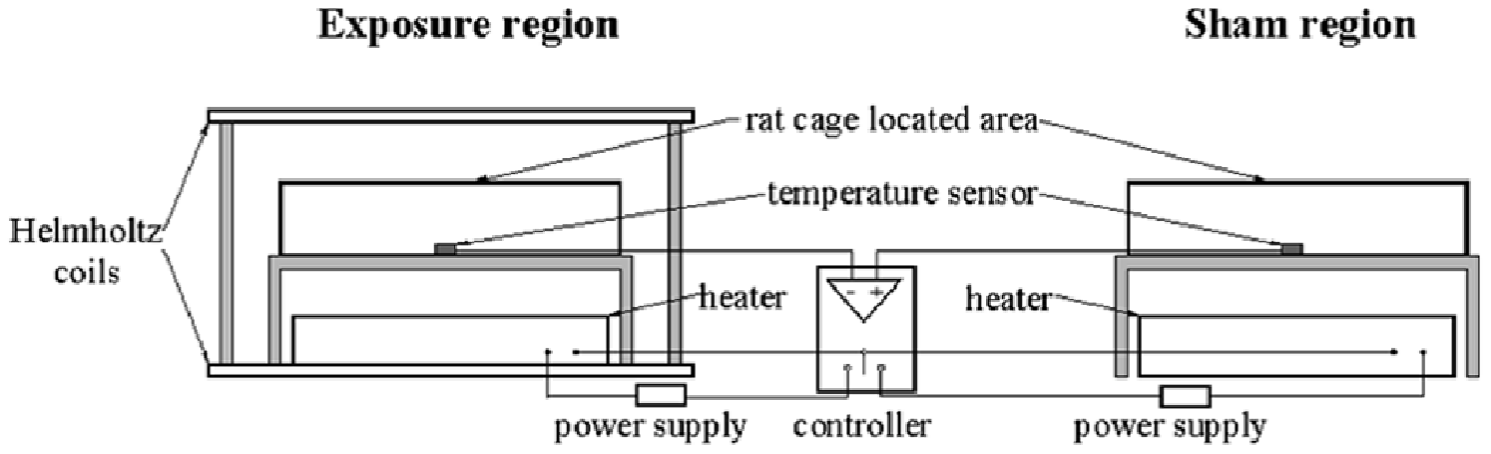
Diagram of the exposure system and field characteristics. MF group and MF+Al group rats were kept in the exposure region which includes a pair of Helmholtz coils. Control group and Al group rats were placed in a geomagnetic environment in the sham region. A temperature control system composed of a controller, two temperature sensors, and two heaters were used to reduce the temperature differences between the two regions.

A temperature control system composed of a controller, two temperature sensors, and two heaters was used to reduce temperature differences between the exposure region and the sham region. Temperature signals in the two regions were received by sensors and transferred to the controller. The heater was programmed to switch on when the temperature of one area became lower than that of the other (Fig. 1). The temperature control system ensured that temperature differences between the exposure and sham region were less than 0.2°C.

The vertical, north-south, and east-west components of the geomagnetic field were measured and found to be 32 µT, 21 µT and 9 µT, respectively, using a fluxgate magnetometer (National Institute of Metrology, Beijing, China).

### Behavioral Testing

After 12 wk of MF exposure and AlCl_3_ solution treatment, the Morris water maze (MWM) test was performed. The MWM (Institute of Materia Medica, Chinese Academy of Medical Sciences, China) is a circular pool (diameter: 150 cm; height: 45 cm) with a flat black inner surface. The pool is surrounded by black curtains with prominent visual cues and filled with tap water maintained at 23°C ±1°C. Black nontoxic ink is added to the pool to make the water opaque. The pool was divided into four quadrants clockwise and the quadrants were named NE, SE, SW, and NW to indicate north-east, south-east, south-west, and north-west, respectively. A black platform (diameter: 9.5 cm) was positioned at the midpoint of the target quadrant and about 1.5 cm below the surface of the water. A video camera was placed above the pool to record the trajectory of rats.

The MWM test was conducted in two phases. In the spatial acquisition phase, four trials per animal per day were performed for 5 d using 4 randomly selected start locations. Each rat was placed in the water facing the pool wall at a fixed position and allowed to swim freely to the escape platform. If the rat did not reach the platform within 120 s, it was placed on the platform for 15 s [24]. The escape latency and total distance traveled were recorded and analyzed through a behavioral software system (Institute of Materia Medica, Chinese Academy of Medical Sciences, China). A probe trial phase was performed on the sixth day after completion of the acquisition task. The hidden platform was removed and each rat was allowed to swim freely for 30 s. Spatial accuracy was revealed by the number of crossings through the target position. During measurement, the behavior software system operator was blinded to the exposure conditions.

### Biochemical Measurements

The cerebral cortex and hippocampus of six rats from each group were dissected, weighed, and homogenized in ice-cold RIPA lysis buffer (Beyotime Institute of Biotechnology, China). Homogenates were then subsequently subjected to amyloid-β protein quantification using an amyloid-β protein radioimmunoassay kit (Puerweiye Biotechnology Co., Ltd., Beijing, China). The Aβ radioimmunoassay method used in this study is sensitive to all forms of Aβ. Cerebral cortex was dried by an electric-heated oven. Al concentrations in the cortex were examined by inductively coupled plasma atomic emission spectrometry (ICP-AES, SPECTRO Analytical Instruments GmbH, USA) at the Analytical and Testing Center, Beijing Normal University, China.

### Brain Morphology Detection

Under deep anesthesia, four rats from each group were transcardially perfused for 10 min with 0.9% saline and then with 4% cold paraformaldehyde in 0.1 M phosphate-buffered saline (PBS) for 20 min. Brain sections were cut 2 mm posterior to the optic chiasm and post-fixed in the same paraformaldehyde solution for 48 h at 4°C. The tissues were then embedded, sectioned at 10 µm, stained with hematoxylin and eosin (H&E), and photographed using a Nikon E600 camera linked to a Leica DM 4000 B microscope. Two independent observers who were blind to the exposure conditions evaluated 12 randomized optical fields of four coronal sections per group (48 optical fields per group) at 400× magnification. The hippocampal CA1 area and cortical region above the hippocampus were selected as areas for analysis. Neurons that had shrunk and swollen cell bodies with eosinophilic cytoplasm were excluded from the counts. Neuronal numbers are reported as averaged numbers per visual field.

### Statistical Methods

SPSS 20.0 was used for statistical analysis. Data of platform crossings in the probe trials phase are expressed as a number of times for each rat in each group, rank sum test was used to compare mean group differences in platform crossings. Other data are expressed as means ± SD and underwent normality test (Shapiro-Wilk test) and homogeneity of variance test (Levene's Test) before statistical analysis. One-way analysis of variance (ANOVA) followed by least-significant difference (LSD) post hoc test was used for the analysis of water intake. Two-way ANOVA with repeated measures using the day and group as variable was used to analyze the body weight of the four groups, the escape latency, and total distance traveled within 5 d of acquisition, LSD post hoc analysis was used for multiple comparisons between groups. One-way ANOVA followed by LSD post hoc test and two-way ANOVA with the Al and MF as variable were used to analyze Al and Aβ concentrations as well as the density of normal neurons in the brain. Due to the small sample size, there may be increasing chances of having type II errors which could influence the reliability of the normality test, Kruskal-Wallis H test followed by the Mann-Whitney U test was further performed to analyze Al and Aβ concentrations as well as normal neurons density in the brain. *P*<0.05 was considered significant in all statistical tests.

## Results

### General States of Rats

**Figure 2:**
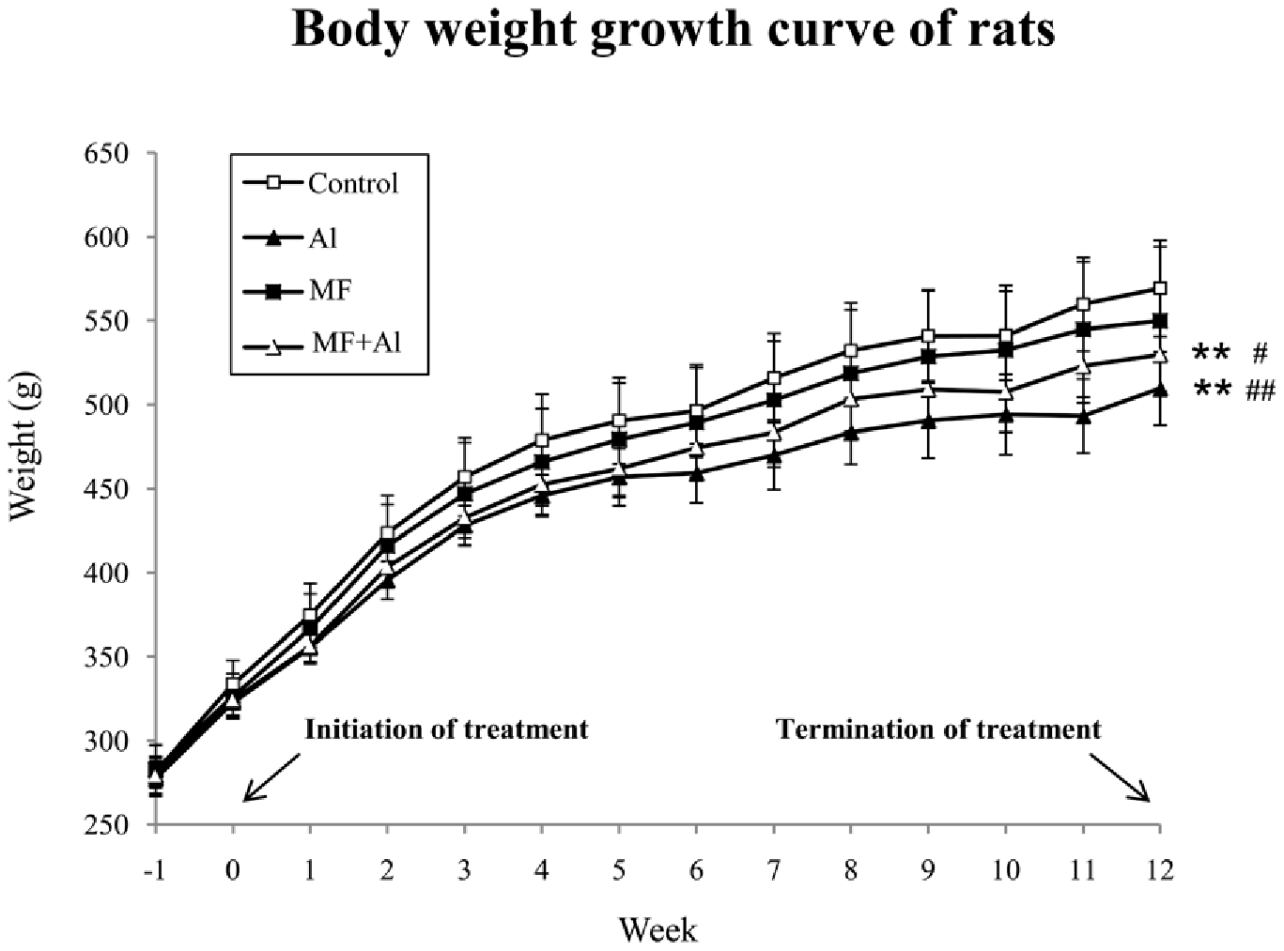
Body weight growth curve of the four groups of rats. The body weights (mean*±*SD) of each group of rats (n = 10 per group) recorded weekly are shown. Two-way ANOVA with repeated measures using the day and group as variable showed that during the 12 wk experiment, differences in body weights between the control and MF groups as well as between the Al and MF+Al groups were not significant. **P<0.01 *vs.* control group, ^#^ *P*<0.05 *vs.* MF group, ^##^ *P*<0.01 *vs.* MF group, n = 10 per group.

After 1 wk of acclimatization, the rats were exposed to ELF-MF and daily oral administration of AlCl_3_ solution at week 0. Body weights were weekly monitored and recorded (Fig. 2). A gradual increase in body weight was observed in all four groups. Two-way ANOVA with repeated measures using the day and group as variable showed that during the 12 wk experiment, the body weights of the Al group rats were lower than those of the control group (*P*<0.01) and MF group (*P*<0.01) rats. The body weights of the MF+Al group rats were significantly suppressed compared with the control group (*P*<0.01) and MF group (*P*<0.05) rats. Differences between the control and MF groups as well as differences between the Al and MF+Al groups were not significant (*P*>0.05).

Table 1 shows ultrapure water and AlCl_3_ solution intake throughout the experiment. The water intake before AlCl_3_ solution treatment were expressed as an average value of 7 d for each rat, during the Al treatment, the water intake were expressed as an average value of 12 w for each rat. No significant differences in the water intakes of all four groups of rats were observed in the 1 wk of acclimatization. During the Al treatment, however, the water intake of the Al group decreased significantly compared with the control group (*P*<0.01) and EMF group (*P*<0.01) rats. The water intake of the MF+Al group also decreased significantly compared with the control group (*P*<0.01) and EMF group (*P*<0.01) rats.

**Table 1:** Ultrapure water and aluminum trichloride solution intake (ml) of rats in each group (n = 10).

	Control	Al	MF	MF+Al
pre-treatment	53.25±5.78	57.50±7.67	55.75±4.64	53.13±8.17
during-treatment	55.04±6.19	32.86±3.71** ##	51.82±5.13	33.75±3.84** ##

The water intake before the AlCl_3_ solution treatment were recorded during the 1 wk of acclimatization and expressed as an average value of 7 d. During the Al treatment, the water intake was expressed as an average value of 12 w for each rat. Two-way ANOVA with repeated measures with the day and group as variable was used. The results showed no significant differences in the water intakes of all four groups of rats before AlCl_3_ treatment. During the Al treatment, no significant differences were observed between the control and MF groups as well as between the Al and MF+Al groups.

** *P*<0.01 *vs.* Control group,

## *P*<0.01 *vs.* MF group.

### Spatial Cognition Changes in MWM Test

**Figure 3:**
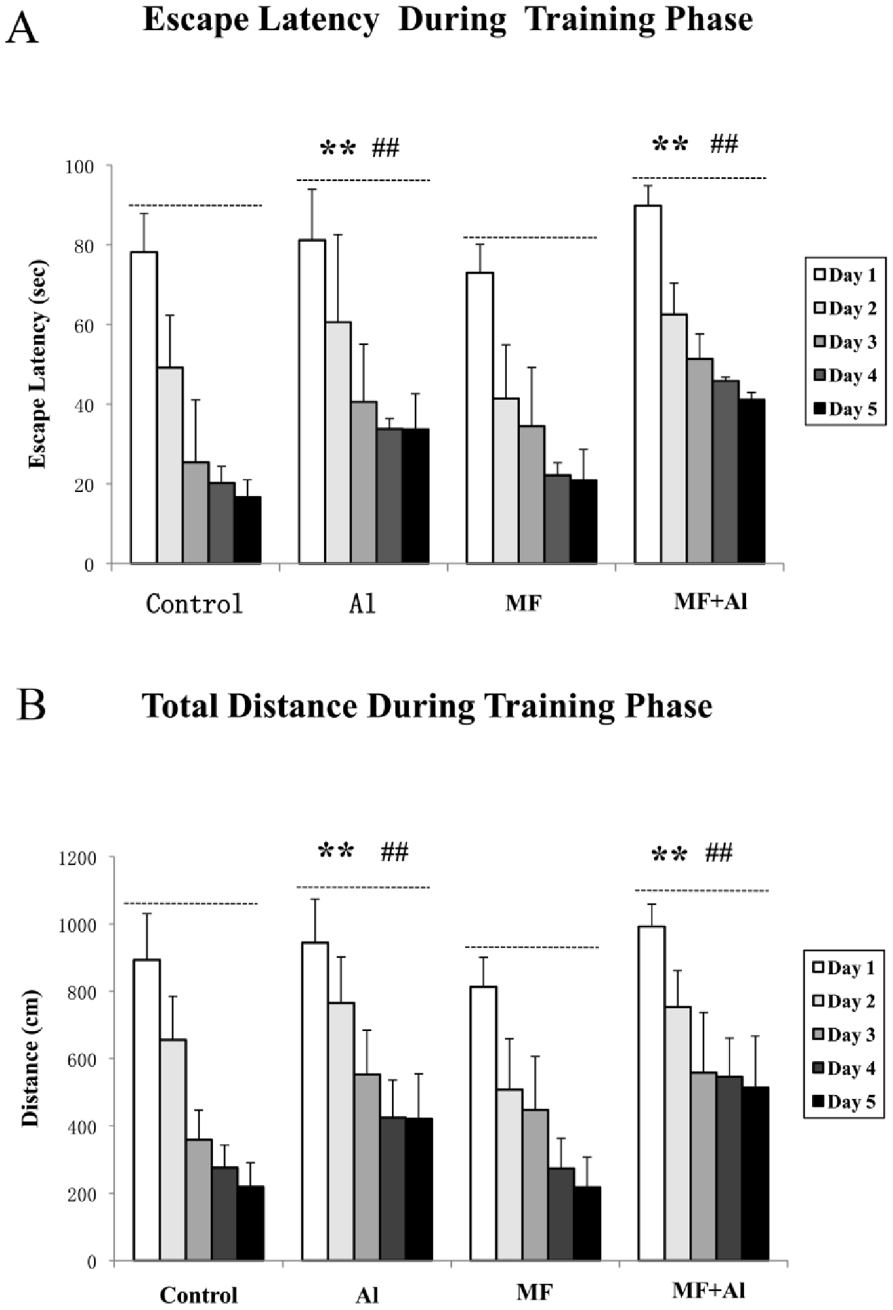
Effects of ELF-MF on escape latency and total distance as measured by the MWM test. In the spatial acquisition phase, four trials per animal per day were performed for 5 d. Two-way ANOVA with repeated measures using the day and group as variable was conducted, and the performances of 5 d among groups were compared. A. Effects of ELF-MF on the escape latency of each rat in each of five training days. B. Effects of ELF-MF on the total distance travelled by each rat in each of five training days. Statistical results of two-way ANOVA with repeated measures showed no significant differences between the control and MF groups as well as between the Al and MF+Al groups. **P<0.01 *vs.* control group, ^##^ *P*<0.01 *vs.* MF group, n = 10 per group.

During the spatial acquisition stage of the MWM experiments, the mean escape latency and swim distance were calculated for each rat in each of the five training days, two-way ANOVA with repeated statistical analysis was conducted. Statistical results showed longer mean escape latencies in the Al group compared with the control (*P*<0.01) and MF (*P*<0.01) groups. The mean escape latency of the MF+Al group significantly increased compared with that of the control (*P*<0.01) and MF (*P*<0.01) groups. No differences between the control and MF groups as well as between the Al and MF+Al groups (*P*>0.05) (Fig. 3A) were observed. Similar results were obtained for swimming distance (Fig. 3B). During the probe trial stage, data of platform crossings were expressed as a number of times for each rat in each group (Table 2). Rank sum tests of platform crossings confirmed that the rats in the Al group performed significantly worse than those in the control (*P*<0.05) and MF (*P*<0.05) groups. Similarly, MF+Al group rats performed significantly worse than those in the control (*P*<0.05) and MF (*P*<0.05) groups.

**Table 2:** Platform crossings for each rat by group during the probe test.

Groups	Platform crossings (number of times)									
	1	2	3	4	5	6	7	8	9	10
Control	2	1	1	4	0	1	2	0	2	0
Al* #	1	0	0	0	0	1	0	0	0	0
MF	2	1	1	0	0	1	2	1	3	0
MF+Al* #	1	1	0	0	0	0	0	1	0	0

During the probe test at the sixth day after finishing the acquisition task, each rat was allowed to swim freely for 30 s, the number of crossings through the target position was recorded for each rat by group (10 rats per group). Rank sum tests revealed no significant differences in platform crossings between the Al and MF+Al groups and between the control and MF groups.

* *P*<0.05 vs. Control group,

# *P*<0.05 vs. MF group, n = 10 per group.

### Al Content and Aβ Concentration in the Brain Tissue

**Figure 4:**
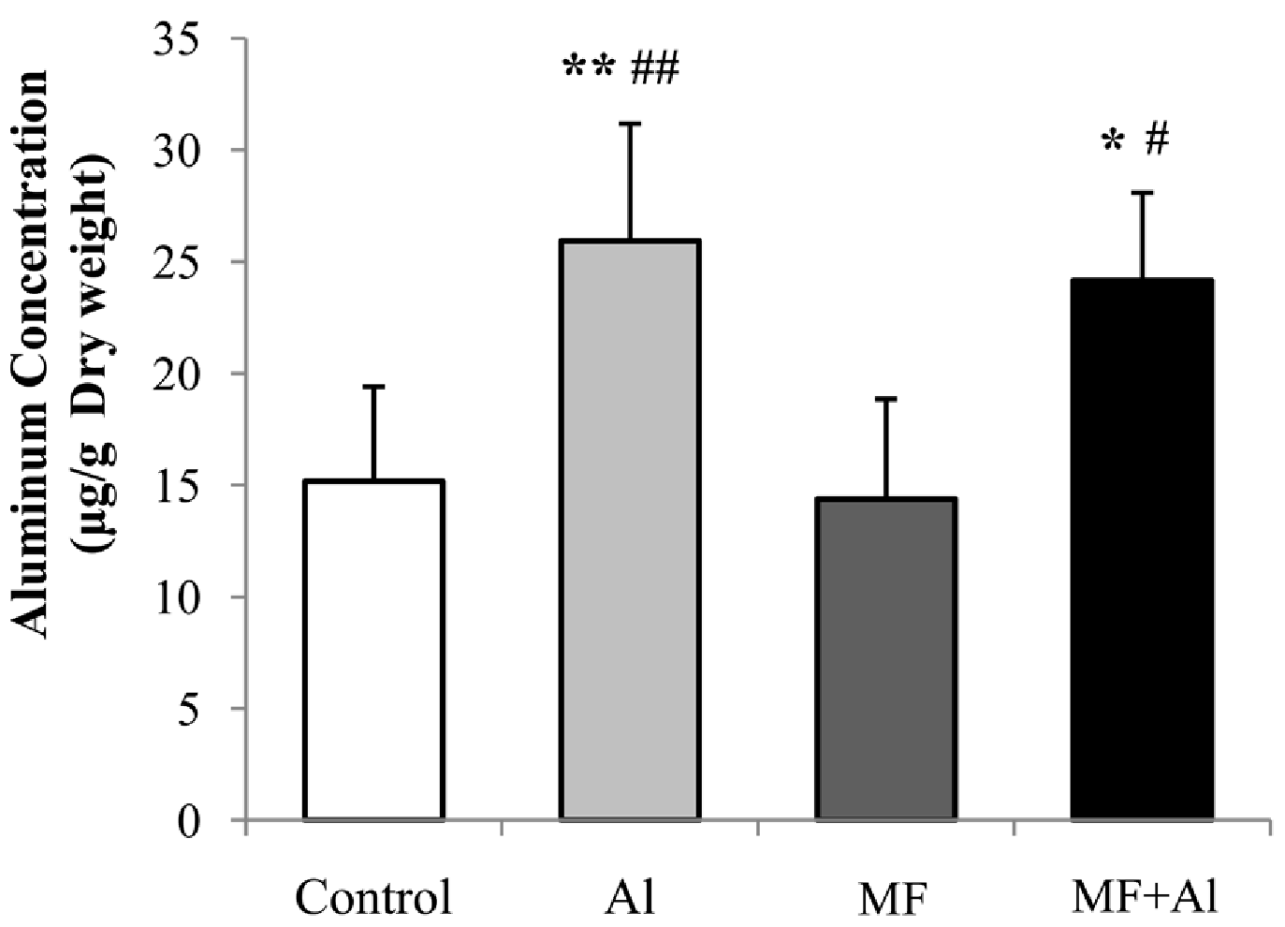
Al levels in dry cerebral cortex determined by ICP-AES. One-way ANOVA followed by LSD post hoc test showed no significant differences in Al levels between the control and MF groups as well as between the Al and MF+Al groups in the cerebral cortex. The same statistical analysis results were obtained by using Kruskal-Wallis H test followed by the Mann-Whitney U test. *P<0.05 *vs.* control group, **P<0.01 *vs.* control group, ^#^ *P*<0.05 *vs.* MF group, ^##^ *P*<0.01 *vs.* MF group, n = 6 per group.

One-way ANOVA followed by LSD post hoc test showed no significant differences in Al levels between the control and MF groups as well as between the Al and MF+Al groups (*P*>0.05). The Al concentration in the cortices of the Al group rats significantly increased compared with those of the control (*P*<0.01) and MF (*P*<0.01) group rats. Significantly greater accumulation of Al was observed in the cortices of the MF+Al group rats compared with those in the control (*P*<0.05) and MF (*P*<0.05) group rats, as shown in Fig. 4. The statistical analysis results of Kruskal-Wallis H test followed by the Mann-Whitney U test were the same as the results of one-way ANOVA followed by LSD post hoc test.

**Figure 5:**
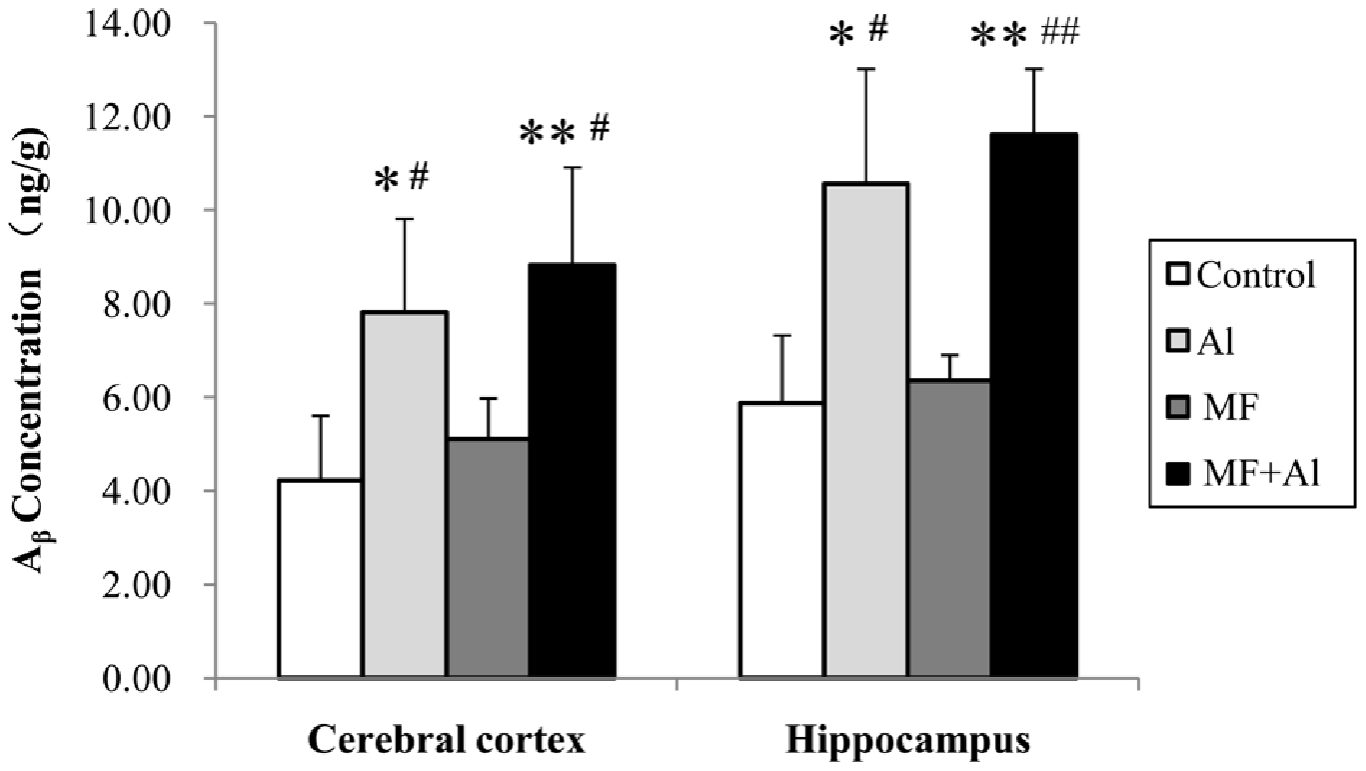
Amounts of amyloid-β (Aβ) deposited in the hippocampus and cerebral cortex. One-way ANOVA followed by LSD post hoc test showed no significant differences were observed in terms of Aβ concentration in the cortex and hippocampus between the control and MF groups as well as between the Al and MF+Al groups. The same statistical analysis results were obtained by using Kruskal-Wallis H test followed by the Mann-Whitney U test. *P<0.05 *vs.* Control group, **P<0.01 *vs.* Control group, ^#^ *P*<0.05 *vs.* MF group, ^##^ *P*<0.01 *vs.* MF group, n = 6 per group.

One-way ANOVA followed by LSD post hoc analysis of cerebral cortical tissues showed that compared with the control group, Aβ concentration significantly increased both in the Al (*P*<0.05) and MF+Al (*P*<0.01) groups (Fig. 5). Compared with the MF group, Aβ concentration significantly increased in the Al (*P*<0.05) and MF+Al (*P*<0.05) groups. Similar results were observed in terms of hippocampal tissues. Aβ deposited in the hippocampus of the Al (*P*<0.05) and MF+Al (*P*<0.01) group rats was higher than that of the control group rats. Compared with the MF group, Aβ concentrations significantly increased in the Al (*P*<0.05) and MF+Al (*P*<0.01) groups (Fig. 5). No significant differences between the control and MF groups as well as between the Al and MF+Al groups with respect to Aβ concentration in the cortex and hippocampus were observed (*P*>0.05) (Fig. 5). The same statistical analysis results were obtained by using Kruskal-Wallis H test followed by the Mann-Whitney U test.

Two-way ANOVA using the factors MF and Al were further conducted. Statistical results showed that oral Al treatment (*P*<0.01), rather than ELF-MF exposure (*P*>0.05), significantly influences Al content and Aβ concentration in the brain tissue. No combined effects were found between MF exposure and oral Al treatment (*P*>0.05).

### Brain Morphology and Neural Loss

**Figure 6:**
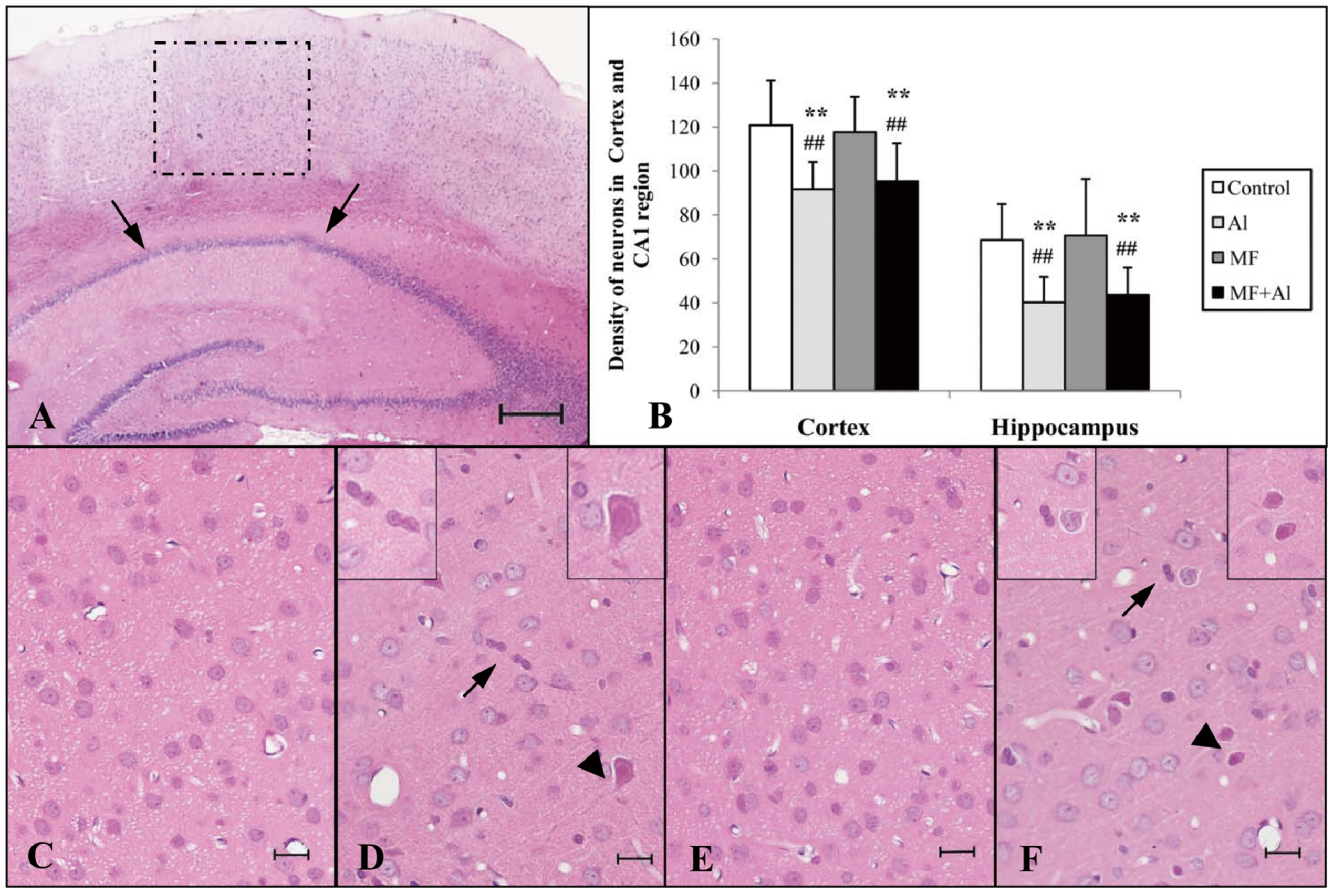
H&E staining results of the cerebral cortex and hippocampus. (A) The hippocampal CA1 area (arrows) and cortical region above the hippocampus (dashed box) were used for analysis (magnification, 400×). (B) The histogram demonstrates the density of normal neurons in the cerebral cortex and hippocampus CA1 area. One-way ANOVA followed by LSD post hoc test showed no significant differences in terms of densities of normal neurons in the cerebral cortex between the Al and MF+Al groups as well as between the control and MF groups. Quantitative results of hippocampal neurons were similar to those of the cerebral cortex. Differences between the control and MF groups as well as between the Al and MF+Al groups were not significant. The same statistical analysis results were obtained by using Kruskal-Wallis H test followed by the Mann-Whitney U test. **P<0.01 *vs.* control group, ^##^ *P*<0.01 *vs.* MF group, n = 6 per group. (C) Control group and (E) MF group: normal morphology with few shrunken neurons. (D) Al group and (F) MF+Al group: normal neurons with distinguishable cell membranes and clear nucleoli close to shrunken (arrows) and swollen (arrowheads) neurons. Bar = 200 µm in A. Bar = 10 µm in C, D, E, F, n = 48 per group.

The number of neurons in the cortex and hippocampus was assessed from H&E-stained brain slices (10 µm), as shown in Fig. 6. The areas used for counting H&E-positive neurons are demonstrated in panel A in the figure, and the hippocampal CA1 area (arrow) as well as the cortical region above the hippocampus (dashed box) were selected for analysis.

Panel C (control group) exhibited normal corticular neuron morphology with distinguishable cell membranes and clearly nucleoli. Similar results were observed in the MF group cortex (Panel E). By contrast, the cerebral cortex of the Al (Panel D) and MF+Al (Panel F) groups showed a shrunken (arrows) and swollen (arrowheads) neurons with eosinophilic cytoplasm. Abnormal neurons were excluded from counting during the determination of neuron density.

Quantitative evaluations of the histological results are shown in Panel B, the density of normal neurons in the brain underwent one-way ANOVA followed by LSD post hoc test. The density of normal cerebral corticular neurons in the Al group significantly decreased compared with those in the control (*P*<0.01) and MF (*P*<0.01) groups. Similarly, the density of cerebral corticular neurons in the MF+Al group (*P*<0.01) significantly decreased compared with those in the control (*P*<0.01) and MF (*P*<0.01) groups. No significant differences were found between the Al and MF+Al groups (*P*>0.05) and between the control and MF groups (*P*>0.05). Quantitative results of hippocampal neurons were similar to those of the cerebral cortex. Al caused a decrease in the densities of normal neurons in the Al and MF+Al groups compared with those in the control (*P*<0.01) and MF (*P*<0.01) groups. However, differences between the control and MF groups as well as between the Al and MF+Al groups were not significant (*P*>0.05). The same statistical analysis results were obtained by using Kruskal-Wallis H test followed by the Mann-Whitney U test.

Furthermore, according to the results of two-way ANOVA using the factors MF and Al, Al treatment significantly decreased neuron densities in the Al and MF+Al groups. However, significant effects of MF alone and MF+Al were not observed (*P*>0.05).

## Discussion

The magnetic flux density (100 µT) used in this study is the public 50 Hz MF exposure limit applied by many countries to protect the health of the general population [25]. Parameters such as exposure limits for public health are typically very conservative. At present, scientific evidence related to the possible health effects of long-term, low-level exposure to ELF fields remains insufficient [26]. This research studied the long-term effects of ELF-MF exposure on AD pathogenesis in Al-overloaded rats. Our findings represent the first step toward investigating the long-term effects of ELF-MF exposure on animals.

According to the primary clinical symptoms of AD, which include learning and memory impairment, we detected the cognition of four groups of rats through MWM tests. The biological effect of weak magnetic exposure on learning and memory remains disputed [27]–[29]. The experimental conditions in the present study were strictly controlled to avoid external conditions from influencing our findings. The results showed that 12 wk of chronic exposure to MF did not significantly impede learning and memory ability, which may be due to the low field strength of the ELF-MF. Fu *et al.* [27] found that short-term exposure to ELF-MF (1.1 mT at 50 Hz) has no significant effect on the Y-maze performance of rats; however, long-term exposure (2 mT at 50 Hz) reduces the ability of the rats for recognition. Another study regarding dose-response effects was reported by Sienkiewicz, who indicated that exposure to 0.75 mT and 7.5 mT fields significantly impairs the performance of mice in an eight-arm radial maze; however, the difference between the control group and groups exposed to 75 µT MF was not significant [29]. This result is consistent with our experimental findings.

In addition to impairments in progressive learning and memory, AD is also characterized by morphological hallmarks such as neuronal cell loss and high density of senile plaques in the brain [3], [30], [31]. We respectively detected normal neuron density and Aβ content, which is the main component of amyloid plaques, in the cortex and hippocampal CA1 area. The results further confirm that long-term ELF-MF exposure (100 µT at 50 Hz) does not influence AD pathogenesis.

Some studies have questioned the relationship between Al and AD [32], [33] while numerous others continue to emphasize the relation between the two in which Al-induced brain changes have been well documented [34]–[37]. Luo *et al.* [34] demonstrated that chronic application of Al causes high Aβ levels in the mouse cortex and hippocampus. Al accumulation in the brain after oral Al treatment for 6 m was reported by Fattoretti *et al.* [33]. Similar results were observed in Al-overloaded rats in the present study, with high levels of Aβ deposition and increased cortical Al concentrations in the brain. In some perspectives, the Al group may also be considered as a positive control and the results further prove the reliability of the experiment. To the best of our knowledge, this study is the first report investigating the combined impact of Al treatment and magnetic exposure on the pathogenesis of AD. The results demonstrate that the combined treatment of MF exposure and Al overdose does not result in increased Al and Aβ concentrations compared with Al treatment alone in the brain.

Bhasin [38] reported that Al treatment (AlCl_3_, 100 mg/kg b.wt./day) causes signs of oxidative stress and liver damage in rats. However, in the present study, the results of swim speed demonstrated no significant differences between the control group and Al group rats. Therefore, no clear evidence that impaired performance in the water maze is secondary to swim speed impairment is available. The MWM results were reliable in assessing the learning and memory of SD rats in our experiment. Apparent reductions in the water intake of the Al-treated groups may be due to the taste of the AlCl_3_ solution (the pH of AlCl_3_ solution is about 4.0; that of ultrapure water is about 7.0). Suppression of the body weight gain of the Al and MF+Al group rats indicated that the Al oral treatment, rather than ELF-MF exposure, negatively influences body weight gain.

Previous studies have shown that ELF-MF may be involved in neurodegenerative processes by influencing calcium ion homeostasis at the cellular level [39], [40] or by directly reacting with specific DNA sites at the gene level [41]. Future studies must be carefully designed to examine the effects of different ELF-MF exposure levels on AD pathogenesis and provide further insights into the effects of ELF-MF on the development of AD at the cellular and gene levels.
